# A cross-sectional study on the relationship between dietary magnesium intake and periodontitis in different body mass index and waist circumference groups: National Health and Nutrition Examination Survey, 2009–2014

**DOI:** 10.1017/jns.2025.19

**Published:** 2025-05-08

**Authors:** Huijie Huang, Shiyan Chen, Li Cong, Yingjuan Zeng

**Affiliations:** 1 Department of Endocrinology and Metabolism, The First Huizhou Affiliated Hospital, Guangdong Medical University, Huizhou, China; 2 Department of Endocrinology and Metabolism, The Fifth Affiliated Hospital, Sun Yat-sen University, Zhuhai, China

**Keywords:** Body mass index, Dietary magnesium intake, Obesity, Periodontitis, Waist circumference, NHANES, National Health and Nutrition Examination Survey, BMI, Body mass index, WC, Waist circumference, CVD, Cardiovascular diseases, DM, Diabetes mellitus, WHO, World Health Organization, AL, Attachment loss, PD, Probing depth, CDC-AAP, Centers for Disease Control and Prevention and the American Academy of Periodontology, AMPM, Automated Multiple Pass Method, FNDDS, Food and Nutrient Database for Dietary Studies, OR, Odds ratios, CI, Confidence interval

## Abstract

This cross-sectional study aimed to investigate the correlation between magnesium consumption and periodontitis in different body mass index (BMI) and waist circumference (WC) groups. 8385 adults who participated in the National Health and Nutrition Examination Survey during 2009–2014 were included. The correlation between dietary magnesium intake and periodontitis was first tested for statistical significance by descriptive statistics and weighted binary logistic regression. Subgroup analysis and interaction tests were performed to investigate whether the association was stable in different BMI and WC groups. There was a statistical difference in magnesium intake between periodontitis and non-periodontitis populations. In model 3, participants with the highest magnesium consumption had an odds ratio of 0.72 (0.57-0.92) for periodontitis compared to those with the lowest magnesium consumption. However, in subgroup analysis, the relationship between magnesium intake and periodontitis remained significant only in the non-general obese (BMI ≤ 30 kg/m^2^) and non-abdominal obese populations (WC ≤ 102 cm in men and ≤ 88 cm in women). Dietary magnesium intake might decrease the periodontitis prevalence in the American population, and this beneficial periodontal health role of magnesium consumption might only be evident in non-general obese and non-abdominal obese populations.

## Introduction

Periodontitis is a non-communicable disease induced by dental plaque and host-mediated inflammatory destruction of tooth-supporting tissues^(1)^, with a prevalence of 50% in China and 40% in the United States, making it the sixth most common disease^(2,3)^. A substantial body of evidence links periodontitis to several systemic diseases^(4)^ such as atherosclerotic disease^(5)^, cardiovascular diseases (CVD)^(6,7)^, diabetes mellitus (DM)^(8,9)^, rheumatic disease^(10)^, Alzheimer’s disease^(11)^, and chronic lower respiratory diseases^(12)^. Moreover, periodontal therapy may improve systemic disease outcomes in patients with periodontitis, for example, by improving arterial stiffness for its beneficial effects on flow-mediated dilatation and carotid intima-media thickness^(13)^, and lowering glycated haemoglobin in diabetic patients^(14)^, resulting in a growing interest in periodontitis.

Nutrients derived from the diet are essential for lifelong health and development by providing a vital energy source and essential cofactors required for enzymes to function, structural moieties, and transport^(15)^. Magnesium, one of the nutrients, plays an important role in regulating oxidative stress and inflammatory responses^(16)^. Studies support the statement that the deficiency of magnesium may have a significant contribution to the occurrence of periodontitis^(17–19)^ and systemic diseases^(20–25)^. An investigation involving 4290 participants indicated that the periodontal health of subjects taking magnesium-containing drugs was improved compared with nonusers of magnesium-containing drugs^(26)^. Therefore, deterioration of periodontal health should be viewed as an early warning sign to control the quality of the patient’s diet in order to reduce the risk of developing systemic diseases later in life. These suggestions may have significance in obesity, which is characterised as a chronic low-grade inflammation component, and shares a comorbidity effect with common non-communicable diseases^(27)^. However, the relationships between magnesium, obesity, and periodontitis are uncertain. To our knowledge, there was limited information available on whether the correlation between dietary magnesium intake and oral health remains consistent in non-obese and obese populations. Therefore, the current study analysed the data from the National Health and Nutrition Examination Survey (NHANES, 2009–2014) aiming to evaluate the potential association between dietary magnesium intake and periodontitis in the obese population which would be defined by body mass index (BMI) and waist circumference (WC).

## Methods

### Study population

**Fig. 1.**
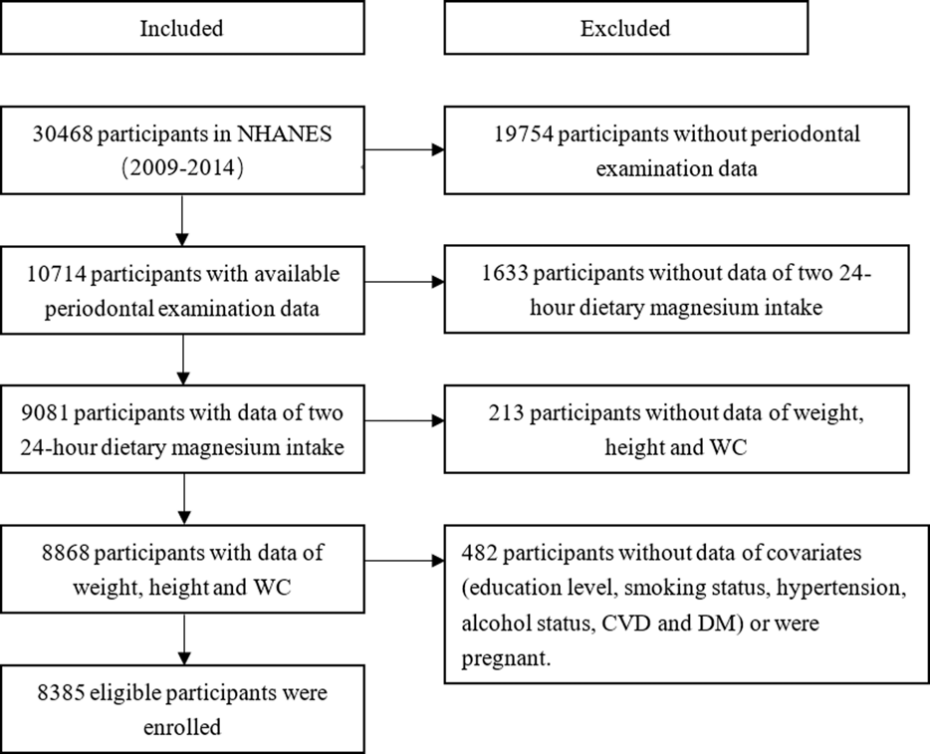
Flowchart for the selection of eligible participants. Abbreviation: NHANES, National Health and Nutrition Examination Survey; WC, waist circumference; DM, diabetes mellitus; CVD, cardiovascular disease.

The current study collected data from NHANES, a complex, multistage, probability sampling survey designed to assess the health and nutritional status of the non-institutionalised United States population. The NHANES interview includes demographic, socioeconomic, dietary, and health-related questions. The examination component consists of medical, dental, and physiological measurements, as well as laboratory tests administered by highly trained medical personnel. According to NHANES standards for periodontal exams, only participants who were 30 years of age or older and who retained at least one natural tooth performed periodontal exams. Therefore, the exclusion criteria for this study were (1) people who did not receive periodontal exams (people younger than 30 years of age or without a single natural tooth); (2) people without valid data of two 24-hour dietary magnesium intake; (3) people without weight, height, and WC data; (4) pregnant women; (5) people without data for potential confounders (including age, gender, race, educational attainment, smoke status, alcohol use status, DM, CVD, and hypertension). We selected 30,468 participants from 3 survey cycles over six years (2009–2014). 10714 participants with periodontal examination data were enrolled in the study. Then we excluded 1633 participants without valid data on two 24-hour dietary magnesium intake, 213 participants with missing data on weight, height, and WC, and 482 participants with missing information on potential confounders or who were pregnant, leaving a total of 8385 subjects included in the analysis (Fig. 1).

### Definition of general obesity and abdominal obesity

BMI is calculated by dividing weight in kilograms by height square in metres. In accordance with World Health Organization classification (WHO), cut-off points recommendations for the increased risk of metabolic complications (WHO, 2008), general obesity was characterised by a BMI greater than 30 kg/m^2^, and abdominal obesity was defined as having a WC >102 cm in men and >88 cm in women.

### Definition of periodontal disease

Periodontitis data were obtained from Oral Health-Periodontal in Examination Data of NHANES 2009–2014. Participants aged ≥ 30 years were eligible for a full-mouth periodontal examination if they had ≥1 natural tooth present. In this study, we used the criteria proposed by Eke et al.^(28)^. Two sets of clinical periodontal measurements were included: clinical attachment loss (AL) and probing depth (PD). The Centers for Disease Control and Prevention and the American Academy of Periodontology (CDC-AAP) definition was used to define periodontitis. The classification of periodontitis includes four categories: no, mild, moderate, and severe. Mild periodontitis was characterised by the presence of at least two interproximal sites with AL ≥ 3 mm or more and at least two interproximal sites with PD ≥ 4 mm (not on the same tooth) or one site with PD ≥ 5 mm. Moderate periodontitis was characterised by the presence of at least two interproximal sites with AL ≥ 4 mm (not on the same tooth) or at least two interproximal sites with PD ≥ 5 mm (not on the same tooth). Severe periodontitis was characterised by the presence of at least two interproximal sites with AL ≥ 6 mm (not on the same tooth) and at least one interproximal site with PD ≥ 5 mm. Therefore, no periodontitis was no evidence of mild, moderate, or severe periodontitis. In this study, periodontitis was dichotomised into two groups, present or absent, meaning that participants with mild, moderate, or severe periodontitis were all categorised as having periodontitis.

### Dietary magnesium intake

Trained interviewers conducted two 24-hour dietary recalls to assess total dietary intakes using the Automated Multiple Pass Method (AMPM), a structured interview where participants reported all foods and beverages consumed over the previous 24 hours. The amount of food/beverage consumed is estimated in grams. The magnesium content of foods was estimated using the U.S. Department of Agriculture Food and Nutrient Database for Dietary Studies (FNDDS), which matches reported foods to standardised nutrient values. More details can be found in the Protocol and Procedure of dietary interview in NHANES database. The first 24-hour personal interview was conducted face-to-face in the Mobile Examination Center, and the second was conducted by telephone around 3–10 days later. Dietary magnesium intakes were calculated from the average of data from two dietary recalls. The amount of dietary magnesium intake was separated by quartiles: Q1 (≤ 215.5 mg/d), Q2 (215.6–282 mg/d), Q3 (282.1-368 mg/d) and Q4 (≥ 368.1 mg/d) for data analysis.

### Covariates

Demographic data comprised age (30–59 and ≥ 60 years), gender (male, female), race/ethnicity (White, Black, Mexican American, Other Hispanic, Other Race), and education level (Less Than High School, and High School and above). Smoking status was classified as never smokers, current smokers, and former smokers, based on the answers to the question of whether they have smoked at least 100 cigarettes in their lifetime and whether they were currently smoking. Alcohol status was categorised into five groups: never drinkers (less than 12 drinks throughout their lifetime), former drinkers (more than 12 drinks in their lifetime but not in the past year), mild drinkers (an average intake of no more than 1 drink per day for women and 2 drinks per day for men), moderate drinkers (an average intake of no more than 2 drinks per day for women and 3 drinks per day for men, with ≥ 2 binge drinking days per month) and heavy drinkers (an average of 3 or more drinks per day for women and 4 or more drinks per day for men, with 5 or more binge drinking days per month). DM, CVD, and hypertension were classified based on self-reported.

### Statistical analysis

The statistical analysis was conducted in accordance with guidelines from the Centers for Disease Control and Prevention (https://wwwn.cdc.gov/nchs/nhanes/tutorials/default.aspx). Given the complex probabilistic clustering design of NHANES, which assigns individual sample weights to each respondent, weights were accounted for in all statistical analyses in this study. For the dietary recall interviews, the dietary data are weighted using the respective dietary weight (WTDR2D) for each cycle. Given the proportions of each cycle in the survey design, the weight for each cycle is calculated as 2/6 * WTDR2D. We analysed the data using appropriate sampling weights (1/3 * WTDR2D) to account for the complex survey design used in the NHANES survey.

Continuous variables were converted to categorical variables. The categorical variables are reported as weighted frequencies and weighted percentages. Survey-weighted chi-squared tests were utilised to assess differences of participants with and without periodontitis. Weighted logistic regression was applied to estimate odds ratios (OR) and a corresponding 95% confidence interval (CI) for the associations of dietary magnesium intake with periodontitis. Model 1 was unadjusted for covariates, Model 2 was adjusted for age, gender, and race/ethnicity. Model 3 was additionally adjusted by BMI, WC, education level, smoking status, alcohol drinking status, DM, hypertension, and CVD. Furthermore, subgroup analysis was performed to investigate the potential effect of modification of BMI and WC on the association between magnesium consumption and periodontitis.

The statistical analysis was performed using R version 4.2.1 and the NHANES R package, and p < 0.05 was considered statistically significant.

## Results

### The characteristics of the study population

Table 1 compares the main characteristics of the enrolled subjects based on with or without periodontitis in this study. The prevalence of periodontitis was 49.91%. Compared to the non-periodontitis group, subjects in the periodontitis group were more likely to be older, male, non-Hispanic white, and educated below high school. Participants with general or abdominal obesity were more prone to periodontitis. Those who were never smokers and mild/moderate alcohol users had a lower proportion of periodontitis. Participants with a lower magnesium intake, DM, CVD, and hypertension had a significantly higher prevalence of periodontitis.

**Table 1.** Descriptive characteristics of the study population stratified by periodontitis

Characteristic	Total	Non-periodontitis	Periodontitis	p-value	
	(N = 8385)	(N = 4200)	(N = 4185)		
	n (%)	n (%)	n (%)		
Age				<0.001	
30–59 years	5594(66.71)	3175(79.72)	2419(63.70)		
≥ 60 years	2791(33.29)	1025(20.28)	1766(36.30)		
Gender				<0.001	
Female	4233(50.48)	2483(56.75)	1750(41.81)		
Male	4152(49.52)	1717(43.25)	2435(58.19)		
Race				<0.001	
Mexican American	1164(13.88)	427(5.59)	737(10.97)		
Non-Hispanic black	1714(20.44)	689(7.91)	1025(14.21)		
Non-Hispanic white	3823(45.59)	2187(75.07)	1636(61.89)		
Other Hispanic	830(9.9)	410(4.78)	420(6.06)		
Other races	854(10.18)	487(6.66)	367(6.87)		
Education level				<0.001	
Less than high school	697(8.31)	198(2.38)	499(7.14)		
High school and above	7688(91.69)	4002(97.62)	3686(92.86)		
BMI				0.02	
< 30 kg/m^2^	5069(60.45)	2602(64.16)	2467(60.49)		
≥ 30 kg/m^2^	3316(39.55)	1598(35.84)	1718(39.51)		
WC				0.01	
Normal	3396(40.5)	1747(42.75)	1649(38.29)		
High	4989(59.5)	2453(57.25)	2536(61.71)		
Magnesium intake				0.01	
Q1	2102(25.07)	947(19.82)	1155(24.00)		
Q2	2096(25)	1061(23.86)	1035(24.33)		
Q3	2094(24.97)	1081(27.11)	1013(26.67)		
Q4	2093(24.96)	1111(29.21)	982(24.99)		
Smoke				<0.001	
Former	2185(26.06)	995(25.27)	1190(28.63)		
Never	4705(56.11)	2717(64.47)	1988(45.56)		
Current	1495(17.83)	488(10.26)	1007(25.80)		
Alcohol User				<0.001	
Former	1461(17.42)	579(11.38)	882(19.26)		
Heavy	1462(17.44)	650(16.12)	812(18.73)		
Moderate	1288(15.36)	760(19.85)	528(15.16)		
Mild	3102(36.99)	1685(42.66)	1417(35.95)		
Never	1072(12.78)	526(9.99)	546(10.90)		
DM	1130(13.48)	403(6.80)	727(13.31)	<0.001	
CVD	710(8.47)	222(4.37)	488(11.28)	<0.001	
Hypertension	3738(44.58)	1587(34.90)	2151(46.95)	<0.001	

Abbreviation: BMI, body mass index; WC, waist circumference; DM, diabetes mellitus; CVD, cardiovascular disease.

### Association between dietary magnesium intake and periodontitis

The correlation between dietary magnesium intake and periodontitis is presented in Table 2. We found that participants with the highest quartile of the dietary magnesium intake were less likely to have the risk of periodontitis compared with the lowest quartile of magnesium intake (OR: 0.71; 95%CI: 0.58-0.86; p < 0.001), and the trend p-value was 0.002 in the unadjusted model. After controlling for covariates (Model 2 and Model 3), participants with the highest magnesium consumption had a 39% and 28% lower risk of periodontitis, respectively (Model 2: OR: 0.61; 95%CI: 0.48-0.77; p < 0.001; Model 3: OR: 0.72; 95%CI: 0.57-0.92; p = 0.009).

**Table 2.** Weighted association between dietary magnesium intake and periodontitis

Magnesium intake	OR (95% CI), p-value			
	Model 1	Model 2	Model 3	
Q1	Reference	Reference	Reference	
Q2	0.84 (0.70, 1.01) 0.068	0.80 (0.65, 0.98) 0.036	0.89 (0.73, 1.08) 0.2	
Q3	0.81 (0.65, 1.01) 0.060	0.76 (0.60, 0.96) 0.023	0.86 (0.68, 1.09) 0.2	
Q4	0.71 (0.58, 0.86) <0.001	0.61 (0.48, 0.77) <0.001	0.72 (0.57, 0.92) 0.009	
P for trend	0.002	<0.001	0.012	

Model 1 unadjusted.

Model 2 adjusted for age, gender and race.

Model 3 adjusted for age, gender, race, body mass index, waist circumference, education level. Smoking status, alcohol drinking status, diabetes, hypertension, and cardiovascular disease.

p < 0.05 indicates statistical significance.

### Subgroup analyses

**Fig. 2.**
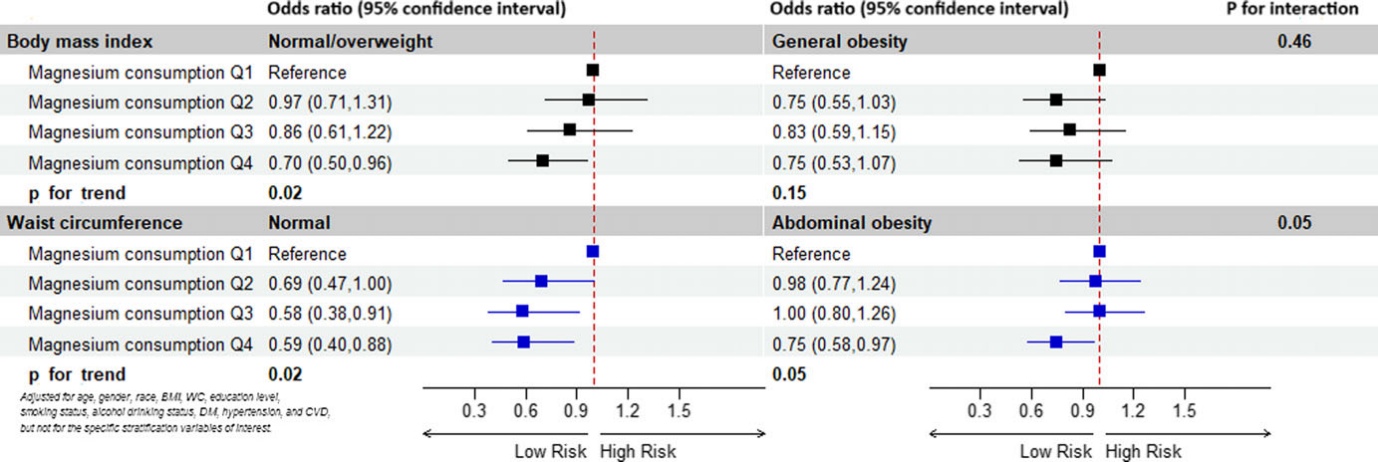
Subgroup analyses for the association between dietary magnesium intake and periodontitis stratified by BMI and WC. Abbreviation: BMI, body mass index; WC, waist circumference; DM, diabetes mellitus; CVD, cardiovascular disease.

Subgroup analyses and interaction tests stratified by BMI and WC were performed to assess whether the relationship between magnesium intake and periodontitis was consistent in the general population and to identify any potentially different population settings (Fig. 2). For participants without general obesity, the higher dietary magnesium intake was associated with periodontitis (p for trend = 0.02). Furthermore, the association was still relevant in subjects without abdominal obesity (p for trend = 0.02). Conversely, in the general obesity and abdominal obesity subgroups, the relationship between dietary magnesium intake and periodontitis was not significant (general obesity: p for trend = 0.15; abdominal obesity: p for trend = 0.05).

## Discussion

The current study revealed a negative association between dietary magnesium intake and periodontitis, but the association was not significant in general obese or abdominal obese populations. The prevalence of periodontitis differed among different socio-demographic segments of the population. The prevalence of periodontitis increases with advancing age. Males, non-Hispanic Whites, and those with general obesity, abdominal obesity, DM, CVD, and hypertension were more likely to suffer from periodontal health problems. Never smokers, mild/moderate alcohol users, and participants with higher education levels had a lower prevalence of periodontitis. The results are consistent with previous studies on periodontitis using the NHANES database^(29–31)^.

The essential function of magnesium in the human body is attributed to its role as a cofactor in more than 300 enzyme systems that regulate a variety of biochemical reactions in the body, including cellular energy metabolism, inflammation, nucleic acid metabolism, protein synthesis, and electrolyte balance^(32)^. Studies confirmed that magnesium was significantly and inversely associated with the concentrations of diverse inflammatory cytokines^(33–36)^. The primary mechanism by which magnesium deficiency has this effect is related to magnesium’s role as a physiologic Ca2+ channel blocker. When magnesium is insufficient, cellular Ca2+ increases, which is the signal that results in the priming of cells to give the inflammatory response^(37)^. Periodontitis, a chronic inflammatory periodontal health condition, has been suggested to be linked to magnesium consumption by a number of studies. Marina et al. verified that a deficiency of magnesium influences periodontitis through systemic loss of bone mass and aggravates inflammatory bone resorption^(38)^. Furthermore, magnesium deficiency has been shown to alter the phenotypic profile of immune cell infiltrate^(18,39)^, suggesting that it might influence immune-mediated tissue destruction in periodontitis. Magnesium, one of the six micronutrients (vitamin A, C, E, selenium, magnesium, and zinc) in the daily diet, was used to calculate dietary antioxidant quality score, and Tianyi et al. analysed data from the NHANES for 2009 to 2014 and found that high-quality group of dietary antioxidant quality score was related to the decreased risk of periodontitis^(17)^. Taking into account that the dietary antioxidant quality score reflects the interactions of multiple nutrients and does not represent the role of a single nutrient, Xin-Yu Li et al. only focused on dietary magnesium intake, treated dietary magnesium intake by quintiles, and confirmed that insufficient magnesium consumption was associated with periodontitis^(19)^. However, the study population was relatively sizable, including only one cycle data from the NHANES, and missed the obesity subgroup when performing stratified analysis. When analysing the relationship between dietary magnesium intake and periodontitis, it is necessary to attach importance to the issue of obesity, since there are different opinions on the relationship between obesity and both magnesium intake and periodontitis.

That magnesium deficiency appeared to be a high-risk factor for obesity was proved in a longitudinal study^(40)^. Comparing the highest to the lowest quintile of dietary magnesium intake, the risk of central obesity was reduced by 18%^(41)^. In Mexican adults, an increase in 10 mg per 1000 kcal/day of magnesium was associated with an average decrease of 0.72% in BMI and 0.49 cm in WC^(42)^. Similarly, several clinical studies have shown that obesity seems to increase the risk of periodontitis^(43)^. It was verified that people with high obesity indices, including BMI, WC, WC-to-height ratio, waist-to-hip ratio, and visceral adiposity index had higher hazards of periodontal disease progression events^(44–47)^. On the contrary, many studies have reached inconsistent conclusions. Studies revealed that the associations between magnesium consumption and body composition indices were meaningless^(48,49)^. In Australian adults, there was a positive association between overweight/weight and periodontitis, but the statistical significance disappeared after adjustment^(50)^. A study of the Fourth Korean National Health and Nutrition Examination Survey led to the conclusion that a high WC seemed to be associated with periodontal infections, whereas BMI was not^(51)^. According to a 4-year study conducted by Tuomas et al. there was no evidence that overweight and obesity can be considered important risk factors in the pathogenesis of periodontitis^(52)^. In consideration of the above conflicting views, further analysis of the correlation between magnesium intake and periodontitis in different BMI and WC groups.

In the current cross-sectional study, we utilised the data of three survey circles from the NHANES (2009–2014) and applied the procedures for participant selection and inclusion, resulting in a total of 8385 participants enrolled for the final analysis. The covariates included age, gender, race, BMI, WC, education level, smoking status, alcohol drinking status, DM, hypertension, and CVD. We came to the same conclusion that the magnesium consumption levels were negatively associated with the prevalence of periodontitis in unadjusted and adjusted models. Furthermore, when performing stratified analysis, this association remained significant only in populations without general obesity or abdominal obesity. One possible reason is that the beneficial role of nutrients appears to be relatively limited when an individual is obese. Obesity signals that the body already has an aggravated systemic inflammation, more severe insulin resistance, and decreased immune function, which is associated with a massive release of a range of inflammatory factors. Adipose tissue secretes a biologically active substance called ‘adipocytokine’, which may be potentially harmful to periodontal tissues and that plasminogen activator inhibitor-1 expressed in visceral fat induces visceral blood coagulation and increases the risk of ischaemic vascular disease, which may also reduce blood flow to the periodontium in obese individuals, and promote periodontal disease^(53)^. The research conducted by Tianyi et al. can support this conjecture. They found that in participants with DM or hyperlipidaemia or both, which are generally considered to be closely related to obesity and chronic inflammation, the association between dietary antioxidant quality score and periodontitis was not significant^(17)^. Another reason may be that individuals with obesity have increased concentrations of cortisol in the blood and urine and that this hormone plays a role in the compartmentalisation of the minerals zinc, selenium, and magnesium, ultimately leading to hypozincemia, hyposelenemia, and hypomagnesemia and increased urinary excretion of these minerals^(54)^. The lower intracellular concentration of magnesium may impair the physiologic function of this nutrient^(55)^. However, these findings still need to be confirmed in future studies.

There are several limitations in the current study: First, as a cross-sectional study, we were unable to determine a causal relationship between magnesium consumption and periodontitis. Second, the data in this study was from the NHANES and applied primarily to the U.S. population. Third, there may be recall bias as the dietary data from NHANES relies on self-reporting. Finally, we cannot completely exclude residual confounding.

### Conclusions

Collectively, these results implied that there was a correlation between dietary magnesium intake and periodontitis in the American population, but this correlation was attenuated in obese populations. This might be related to the fact that magnesium levels decrease in obesity or that the more severe levels of inflammation induced by obesity itself outweigh the anti-inflammatory effects of dietary magnesium. The study emphasised that people should be more aware of lifestyle modification interventions such as healthy diets to successfully tackle multimorbid periodontitis and other chronic diseases.

## Data Availability

All data in this study are available in the NHANES database. This data can be found here: https://www.cdc.gov/nchs/nhanes/index.htm. Further inquiries can be directed to the corresponding author.

## References

[1] Belibasakis GN , Belstrøm D , Eick S , Gursoy UK , Johansson A , Könönen E. Periodontal microbiology and microbial etiology of periodontal diseases: historical concepts and contemporary perspectives. Periodontology. 2000:1–17. 10.1111/prd.12473 36661184

[2] Zhang Q , Li Z , Wang C , et al. Prevalence and predictors for periodontitis among adults in China, 2010. Global Health Action. 2014;7:24503. 25008055 10.3402/gha.v7.24503 PMC4090366

[3] Kwon T , Lamster IB , Levin L. Current concepts in the management of periodontitis. Int Dental J. 2021;71(6):462–476. 10.1111/idj.12630 PMC9275292 34839889

[4] Isola G , Polizzi A , Serra S , Boato M , Sculean A. Relationship between periodontitis and systemic diseases: a bibliometric and visual study. Periodontology. 2000. Published ahead of print, January 8, 2025. 10.1111/prd.12621 PMC12842847 39775963

[5] Herrera D , Molina A , Buhlin K , Klinge B. Periodontal diseases and association with atherosclerotic disease. Periodontology 2000. 2020;83(1):66–89. 32385870 10.1111/prd.12302

[6] Persson GR , Persson RE. Cardiovascular disease and periodontitis: an update on the associations and risk. J Clin Periodontol. 2008;35(8):362–379. 18724863 10.1111/j.1600-051X.2008.01281.x

[7] Kim JY , Lee K , Lee MG , Kim SJ. Periodontitis and atherosclerotic cardiovascular disease. Mol Cells. 2024;47(12):100146. 39515611 10.1016/j.mocell.2024.100146 PMC11612374

[8] Winning L , Patterson CC , Neville CE , Kee F , Linden GJ. Periodontitis and incident type 2 diabetes: a prospective cohort study. J Clin Periodontol. 2017;44(3):266–274. 28036104 10.1111/jcpe.12691

[9] Shinjo T , Nishimura F. The bidirectional association between diabetes and periodontitis, from basic to clinical. Jpn Dent Sci Rev. 2024;60:15–21. 38098853 10.1016/j.jdsr.2023.12.002 PMC10716706

[10] Möller B , Kollert F , Sculean A , Villiger PM. Infectious triggers in periodontitis and the gut in Rheumatoid Arthritis (RA): a complex story about association and causality. Front Immunol. 2020;11:1108. 32582191 10.3389/fimmu.2020.01108 PMC7283532

[11] Ryder MI , Xenoudi P. Alzheimer disease and the periodontal patient: new insights, connections, and therapies. Periodontology 2000. 2021;87(1):32–42. 34463981 10.1111/prd.12389

[12] Sapey E , Yonel Z , Edgar R , et al. The clinical and inflammatory relationships between periodontitis and chronic obstructive pulmonary disease. J Clin Periodontol. 2020;47(9):1040–1052. 32567697 10.1111/jcpe.13334

[13] Polizzi A , Nibali L , Tartaglia GM , Isola G. Impact of nonsurgical periodontal treatment on arterial stiffness outcomes related to endothelial dysfunction: a systematic review and meta-analysis. J Periodontol. 2024:1–16. 10.1002/JPER.24-0422 PMC12062727 39549247

[14] Goyal L , Gupta S , Samujh T. Does nonsurgical periodontal therapy improve glycemic control? Evidence-Based Dentistry. 2023;24(1):21–22. 36890240 10.1038/s41432-023-00860-0

[15] de Baaij JH , Hoenderop JG , Bindels RJ. Magnesium in man: implications for health and disease. Physiol Rev. 2015;95(1):1–46. 25540137 10.1152/physrev.00012.2014

[16] Maier JA , Castiglioni S , Locatelli L , Zocchi M , Mazur A. Magnesium and inflammation: advances and perspectives. Semin Cell Dev Biol. 2021;115:37–44. 33221129 10.1016/j.semcdb.2020.11.002

[17] Zhang T , Hao Y , Zhang R , Lin S. Association between dietary antioxidant quality score and periodontitis: a cross-sectional study. J Dent Sci. 2024;19(1):92–99. 38303792 10.1016/j.jds.2023.05.021 PMC10829658

[18] Malpuech-Brugère C , Nowacki W , Daveau M , et al. Inflammatory response following acute magnesium deficiency in the rat. BBA. 2000;1501(2-3):91–98. 10838183 10.1016/s0925-4439(00)00018-1

[19] Li XY , Wen MZ , Liu H , Shen YC , Su LX , Yang XT. Dietary magnesium intake is protective in patients with periodontitis. Front Nutr. 2022;9:976518. 36091240 10.3389/fnut.2022.976518 PMC9453259

[20] Houston M. The role of magnesium in hypertension and cardiovascular disease. J Clin Hypertens (Greenwich, Conn). 2011;13(11):843–847. 10.1111/j.1751-7176.2011.00538.x PMC8108907 22051430

[21] Dong JY , Xun P , He K , Qin LQ. Magnesium intake and risk of type 2 diabetes: meta-analysis of prospective cohort studies. Diabetes Care. 2011;34(9):2116–2122. 21868780 10.2337/dc11-0518 PMC3161260

[22] Han M , Zhang Y , Fang J , et al. Associations between dietary magnesium intake and hypertension, diabetes, and hyperlipidemia. Hypertens Res: Offic J Japanese Soc Hypertens. 2024;47(2):331–341. 10.1038/s41440-023-01439-z 37821564

[23] Emamat H , Ghalandari H , Totmaj AS , Tangestani H , Hekmatdoost A. Calcium to magnesium intake ratio and non-alcoholic fatty liver disease development: a case-control study. BMC Endocr Disord. 2021;21(1):51. 33736626 10.1186/s12902-021-00721-w PMC7972345

[24] Wang M , Peng J , Yang C , Zhang W , Cheng Z , Zheng H. Magnesium intake and all-cause mortality after stroke: a cohort study. Nutr J. 2023;22(1):54. 37899441 10.1186/s12937-023-00886-1 PMC10614364

[25] Zhang W , Iso H , Ohira T , Date C , Tamakoshi A. Associations of dietary magnesium intake with mortality from cardiovascular disease: the JACC study. Atherosclerosis. 2012;221(2):587–595. 22341866 10.1016/j.atherosclerosis.2012.01.034

[26] Meisel P , Schwahn C , Luedemann J , John U , Kroemer HK , Kocher T. Magnesium deficiency is associated with periodontal disease. J Dent Res. 2005;84(10):937–941. 16183794 10.1177/154405910508401012

[27] Saltiel AR , Olefsky JM. Inflammatory mechanisms linking obesity and metabolic disease. J Clin Invest. 2017;127(1):1–4. 28045402 10.1172/JCI92035 PMC5199709

[28] Eke PI , Page RC , Wei L , Thornton-Evans G , Genco RJ. Update of the case definitions for population-based surveillance of periodontitis. J Periodontol. 2012;83(12):1449–1454. 22420873 10.1902/jop.2012.110664 PMC6005373

[29] Ren Z , Xue Y , Zhang H , et al. Systemic immune-inflammation index and systemic inflammation response index are associated with periodontitis: evidence from NHANES 2009 to 2014. Int Dent J. 2024;74(5):1033–1043. 38688802 10.1016/j.identj.2024.03.019 PMC11561492

[30] Zhao J , Zheng Q , Ying Y , et al. Association between high-density lipoprotein-related inflammation index and periodontitis: insights from NHANES 2009-2014. Lipids Health Dis. 2024;23(1):321. 39342327 10.1186/s12944-024-02312-9 PMC11439298

[31] Wu Y , He B , Chen Q , et al. Association between Mediterranean diet and periodontitis among US adults: the mediating roles of obesity indicators. J Periodontal Res. 2024;59(1):32–41. 37842947 10.1111/jre.13195

[32] Musso CG. Magnesium metabolism in health and disease. Int Urol Nephrol. 2009;41(2):357–362. 19274487 10.1007/s11255-009-9548-7

[33] Rodriguez-Morán M , Guerrero-Romero F. Elevated concentrations of TNF-alpha are related to low serum magnesium levels in obese subjects. Magnesium Res. 2004;17(3):189–196. 15724867

[34] Guerrero-Romero F , Bermudez-Peña C , Rodríguez-Morán M. Severe hypomagnesemia and low-grade inflammation in metabolic syndrome. Magnesium Res. 2011;24(2):45–53. 10.1684/mrh.2011.0281 21609903

[35] Dibaba DT , Xun P , He K. Dietary magnesium intake is inversely associated with serum C-reactive protein levels: meta-analysis and systematic review. Eur J Clin Nutr. 2014;68(4):510–516. 24518747 10.1038/ejcn.2014.7 PMC3975661

[36] Chacko SA , Song Y , Nathan L , et al. Relations of dietary magnesium intake to biomarkers of inflammation and endothelial dysfunction in an ethnically diverse cohort of postmenopausal women. Diabetes Care. 2010;33(2):304–310. 19903755 10.2337/dc09-1402 PMC2809271

[37] Lin CY , Tsai PS , Hung YC , Huang CJ. L-type calcium channels are involved in mediating the anti-inflammatory effects of magnesium sulphate. Br J Anaesthesia. 2010;104(1):44–51. 10.1093/bja/aep336 19933511

[38] Belluci MM , de Molon RS , Rossa C Jr , et al. Severe magnesium deficiency compromises systemic bone mineral density and aggravates inflammatory bone resorption. J Nutr Biochem. 2020;77:108301. 31825817 10.1016/j.jnutbio.2019.108301

[39] Bussière FI , Gueux E , Rock E , et al. Increased phagocytosis and production of reactive oxygen species by neutrophils during magnesium deficiency in rats and inhibition by high magnesium concentration. Br J Nutr. 2002;87(2):107–113. 11895162 10.1079/BJN2001498

[40] Lu L , Chen C , Yang K , et al. Magnesium intake is inversely associated with risk of obesity in a 30-year prospective follow-up study among American young adults. Eur J Nutr. 2020;59(8):3745–3753. 32095867 10.1007/s00394-020-02206-3 PMC7483156

[41] Jiao Y , Li W , Wang L , et al. Relationship between dietary magnesium intake and metabolic syndrome. Nutrients. 2022;14(10):2013. 35631154 10.3390/nu14102013 PMC9144620

[42] Castellanos-Gutiérrez A , Sánchez-Pimienta TG , Carriquiry A , da Costa THM , Ariza AC. Higher dietary magnesium intake is associated with lower body mass index, waist circumference and serum glucose in Mexican adults. Nutr J. 2018;17(1):114. 30518394 10.1186/s12937-018-0422-2 PMC6282375

[43] Suvan J , D’Aiuto F , Moles DR , Petrie A , Donos N. Association between overweight/obesity and periodontitis in adults. A systematic review. Obes Rev: Offic J Int Assoc Study Obes. 2011;12(5):e381–404. 10.1111/j.1467-789X.2010.00808.x 21348914

[44] Gorman A , Kaye EK , Apovian C , Fung TT , Nunn M , Garcia RI. Overweight and obesity predict time to periodontal disease progression in men. J Clin Periodontol. 2012;39(2):107–114. 22150475 10.1111/j.1600-051X.2011.01824.x PMC3258330

[45] Liu L , Xia LY , Gao YJ , Dong XH , Gong RG , Xu J. Association between obesity and periodontitis in US adults: NHANES 2011-2014. Obes Facts. 2024;17(1):47–58. 37935140 10.1159/000534751 PMC10836934

[46] Khader YS , Bawadi HA , Haroun TF , Alomari M , Tayyem RF. The association between periodontal disease and obesity among adults in Jordan. J Clin Periodontol. 2009;36(1):18–24. 19046327 10.1111/j.1600-051X.2008.01345.x

[47] Yang Q , Wang X , Li C , Wang X. A cross-sectional study on the relationship between visceral adiposity index and periodontitis in different age groups. Sci Rep. 2023;13(1):5839. 37037870 10.1038/s41598-023-33082-6 PMC10086006

[48] Guerrero-Romero F , Flores-García A , Saldaña-Guerrero S , Simental-Mendía LE , Rodríguez-Morán M. Obesity and hypomagnesemia. Eur J Intern Med. 2016;34:29–33. 27353277 10.1016/j.ejim.2016.06.015

[49] Mirrafiei A , Jabbarzadeh B , Hosseini Y , Djafarian K , Shab-Bidar S. No association between dietary magnesium intake and body composition among Iranian adults: a cross-sectional study. BMC Nutr. 2022;8(1):39. 35484632 10.1186/s40795-022-00535-6 PMC9052595

[50] Khan S , Bettiol S , Kent K , Barnett T , Peres M , Crocombe LA. Obesity and periodontitis in Australian adults: a population-based cross-sectional study. Int Dent J. 2020;70(1):53–61. 31471898 10.1111/idj.12514 PMC9379158

[51] Kim EJ , Jin BH , Bae KH. Periodontitis and obesity: a study of the Fourth Korean National Health and Nutrition Examination Survey. J Periodontol. 2011;82(4):533–542. 21043799 10.1902/jop.2010.100274

[52] Saxlin T , Ylöstalo P , Suominen-Taipale L , Aromaa A , Knuuttila M. Overweight and obesity weakly predict the development of periodontal infection. J Clin Periodontol. 2010;37(12):1059–1067. 20969609 10.1111/j.1600-051X.2010.01633.x

[53] Saito T , Shimazaki Y. Metabolic disorders related to obesity and periodontal disease. Periodontology 2000. 2007;43:254–266. 17214843 10.1111/j.1600-0757.2006.00186.x

[54] Morais JBS , Cruz KJC , de Oliveira ARS , et al. Association between parameters of cortisol metabolism, biomarkers of minerals (zinc, selenium, and magnesium), and insulin resistance and oxidative stress in women with obesity. Biol Trace Elem Res. 2023;201(12):5677–5691. 37039941 10.1007/s12011-023-03639-7

[55] Dos Santos LR , Melo SRS , Severo JS , et al. Cardiovascular diseases in obesity: what is the role of magnesium? Biol Trace Elem Res. 2021;199(11):4020–4027. 33389619 10.1007/s12011-020-02528-7

